# A case report of two obstructive sleep apnea patients with cervical spine abnormalities and relevant literature review

**DOI:** 10.1002/ccr3.6582

**Published:** 2022-11-27

**Authors:** Anrong Sun, Rui Fang, Huiping Luo

**Affiliations:** ^1^ ENT Institute and Department of Otorhinolaryngology Head & Neck Surgery, The Eye & ENT Hospital Fudan University Shanghai China; ^2^ The Therapy Center of Sleep‐Disordered Breathing, The Eye & ENT Hospital Fudan University Shanghai China

**Keywords:** anatomy abnormalities, cervical spine, continuous positive airway pressure, neck, obstructive sleep apnea

## Abstract

Obstructive sleep apnea (OSA) combined with cervical spine anatomy abnormalities could be an underestimated clinical problem. Here are two cases of this kind reported to draw attention to this issue.

## Graphical abstract

The upper airway compression due to anatomical abnormalities of the cervical spine could be a risk factor of the onset of obstructive sleep apnea.

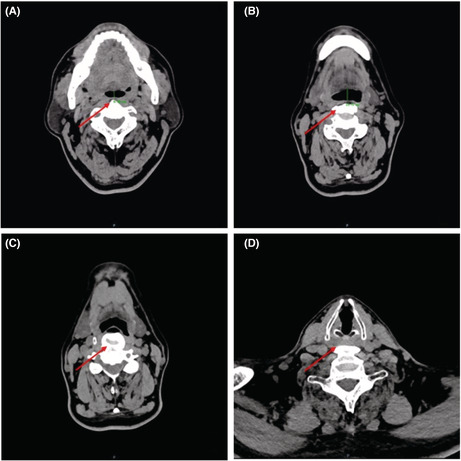

## 1 INTRODUCTION

This article reports two cases of adult patients diagnosed with severe obstructive sleep apnea (OSA) in the Therapy Centre of Sleep‐disordered Breathing of the Eye Ear Nose & Throat (Eye & ENT) Hospital of Fudan University. Both patients were found to have unsuspected cervical spine structural abnormalities. The unexplained correlation between the development of OSA and cervical spine abnormalities inspired our interest.

## 2 CASE REPORT

**FIGURE 1:**
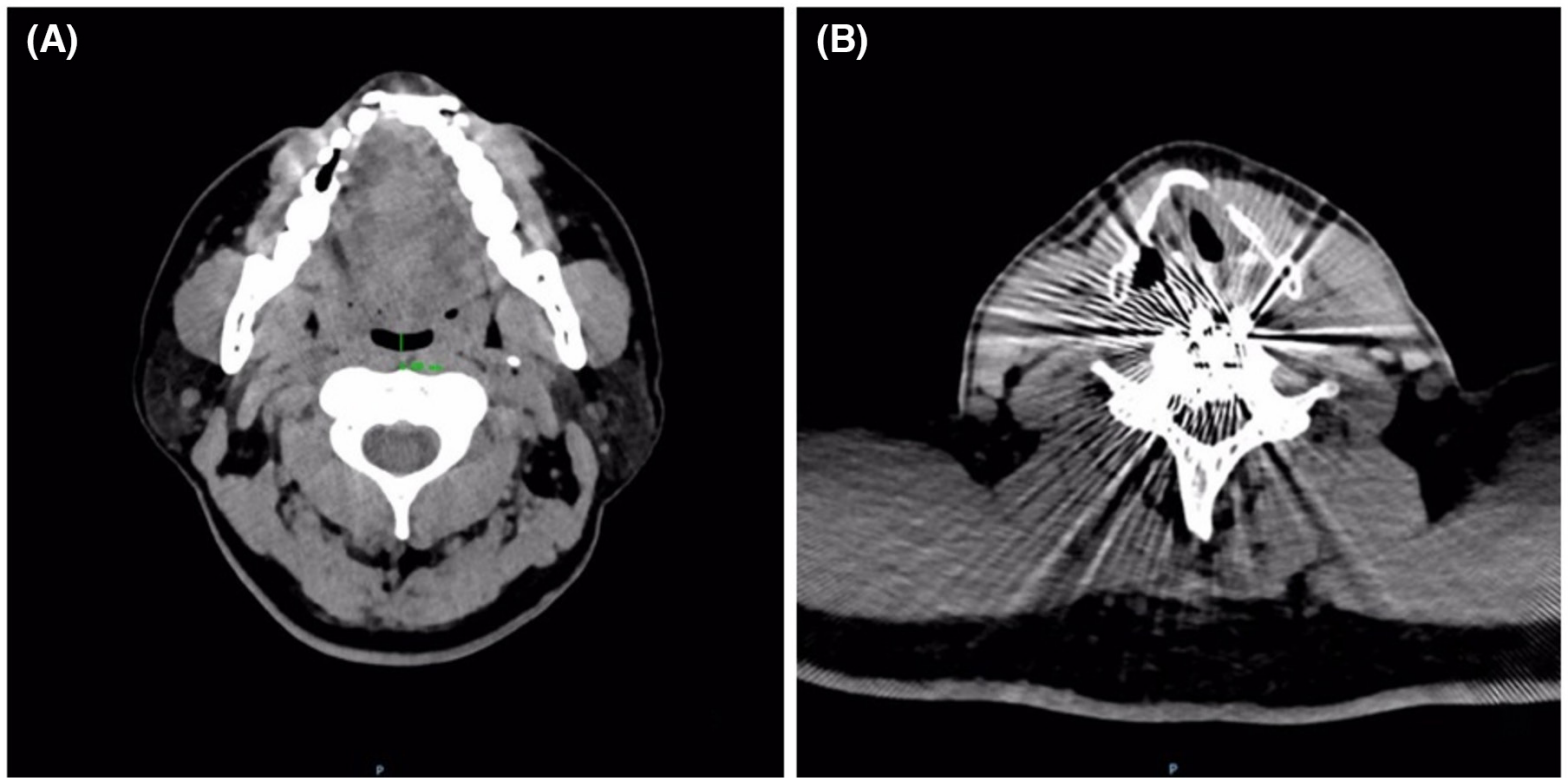
The upper airway computed tomography (UACT) of Case 1. (A) Narrowing of the sagittal diameters (the narrowest point, approx. 4.88 mm) of the oro‐pharyngeal region; according to the data available in China,^6^ the sagittal diameters of this region in the OSA‐free middle‐aged male population is about 13 mm. (B) Post‐operative changes (the layer of glottis) in the cervical spine are shown.

The first patient was a 58‐year‐old man treated from October 26 to October 28, 2020 with complaints of snoring during sleep for 8 years, aggravated with apnea for 3 years. His anthropometric data and physical examination are shown in Table 1. The patient had no history of chronic heart disease, hypertension, type 2 diabetes, or other chronic diseases. He had a history of cervical spondylosis and received the surgical treatment of cervical internal fixation 7 years ago. Upper airway computed tomography (UACT) showed results as follows: (1) Posterior displacement of the soft palate, resulting in narrowing of the sagittal diameters of the oropharyngeal cavity. (2) Postoperative changes in the cervical spine (Figure 1). An overnight polysomnography (PSG) test was performed, and the results (see Table 2) showed that the apnea hypopnea index (AHI) was 44.6/h, the supine AHI (SAHI) was 60.5/h, and the nocturnal minimum oxygen saturation (minSat%) was 83%. The pressure titration was performed for one night, and the 90% treatment pressure of continuous positive airway pressure (CPAP) was 13 cmH_2_O.

**TABLE 1:** Clinical characteristics of these two cases

	Case 1	Case 2
BMI (kg/m^2^)	23.4	23.6
Friedman tongue position	II°	II°
Tonsil grading	I°	I°
Jaw recession	N/A	N/A

*Note*: Friedman tongue position: the patients were told to breathe in a calm state, and the tongue was in a natural position. I°: The entire uvula, tonsils and the post‐pharyngeal wall could be seen. II°: Visualization of the complete soft palate and/or partial uvula and tonsils. III°: Some of the soft palate but not the distal soft palate was seen. IV°: The entire soft palate was hidden, and only the hard palate could be seen. Tonsil grading: Using the Brodsky grading scale, classified into 5 grades: 0°: Post‐tonsillectomy condition. I°: the tonsils were hidden in the pillars. II°: the tonsils were beyond the anterior pillar and between 25% and 50% of the pharyngeal space. III°: the tonsils were beyond the pillars, but not to the middle, and occupied >50% but ≤75% of the pharyngeal space. IV°: the tonsils occupied >75% of the pharyngeal space.

Abbreviation: BMI, Body mass index.

**TABLE 2:** PSG results and CPAP parameters of two cases

	Patient 1	Patient 2
AHI (/h)	44.6	72.5
sAHI (/h)	60.5	74.4
minSat% (%)	83%	81%
AHI after all‐night CPAP (/h)	11	8.8
90% therapeutic pressure of CPAP (cmH_2_O)	13	12

Abbreviations: AHI, apnea hypopnea index; CPAP, continuous positive airway pressure; minSat%, nocturnal minimum oxygen saturation; SAHI, supine apnea hypopnea index.

**FIGURE 2:**
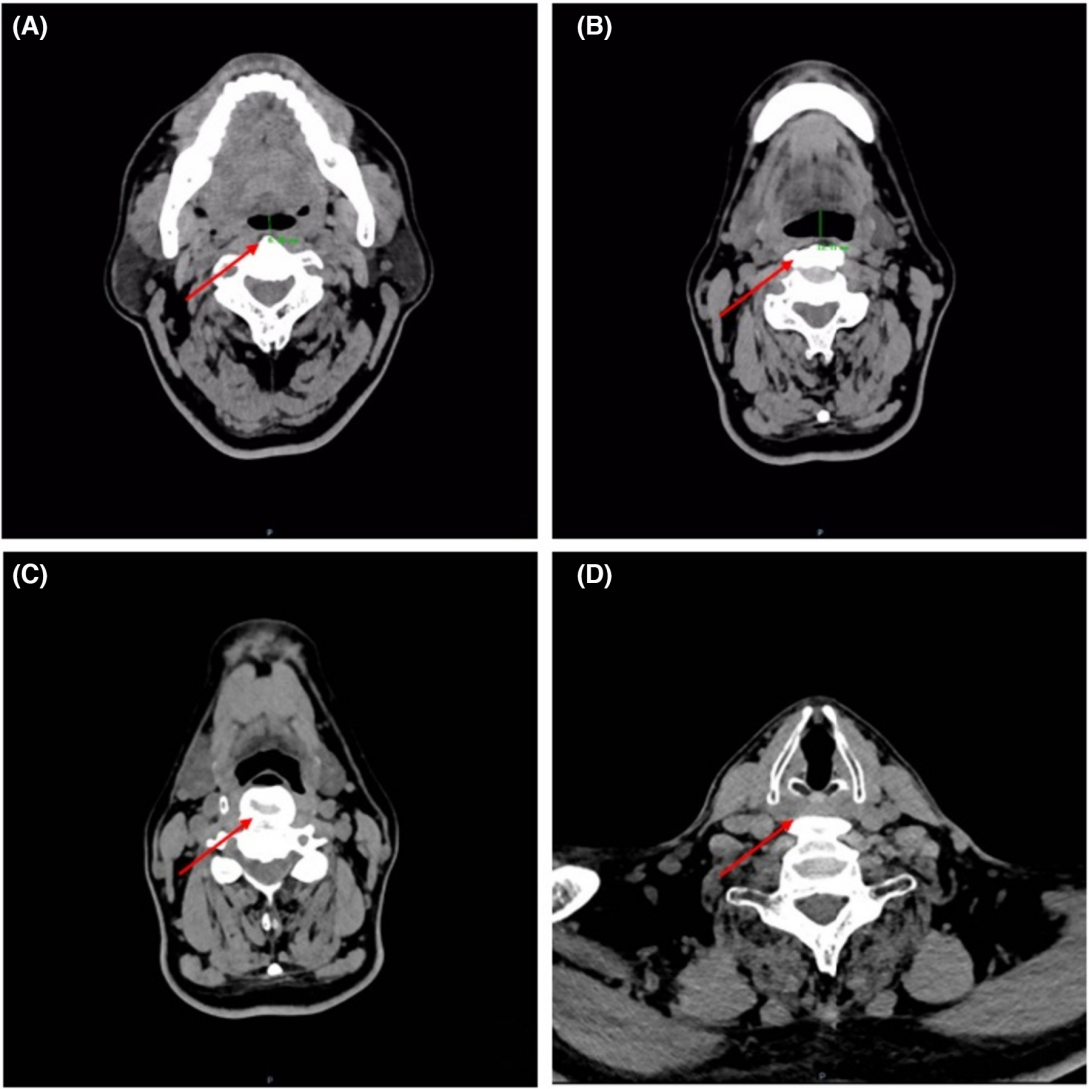
The UACT of Case 2. Obvious osteophytes (where the red arrows point) seen at the anterior edge of the cervical vertebrae resulted in compression of (A) oropharynx, (B, C) larynx‐pharynx and (D) posterior cricoid region. (A) Narrowing of the sagittal diameters of the oropharyngeal cavity (the narrowest point, approx. 6.96 mm) and (B) larynx‐pharyngeal region (the narrowest point, approx. 12.71 mm); according to the data available in China,^6^ the sagittal diameters of these regions in the OSA‐free elderly male population are about 14.29 mm and 16.91 mm.

**FIGURE 3:**
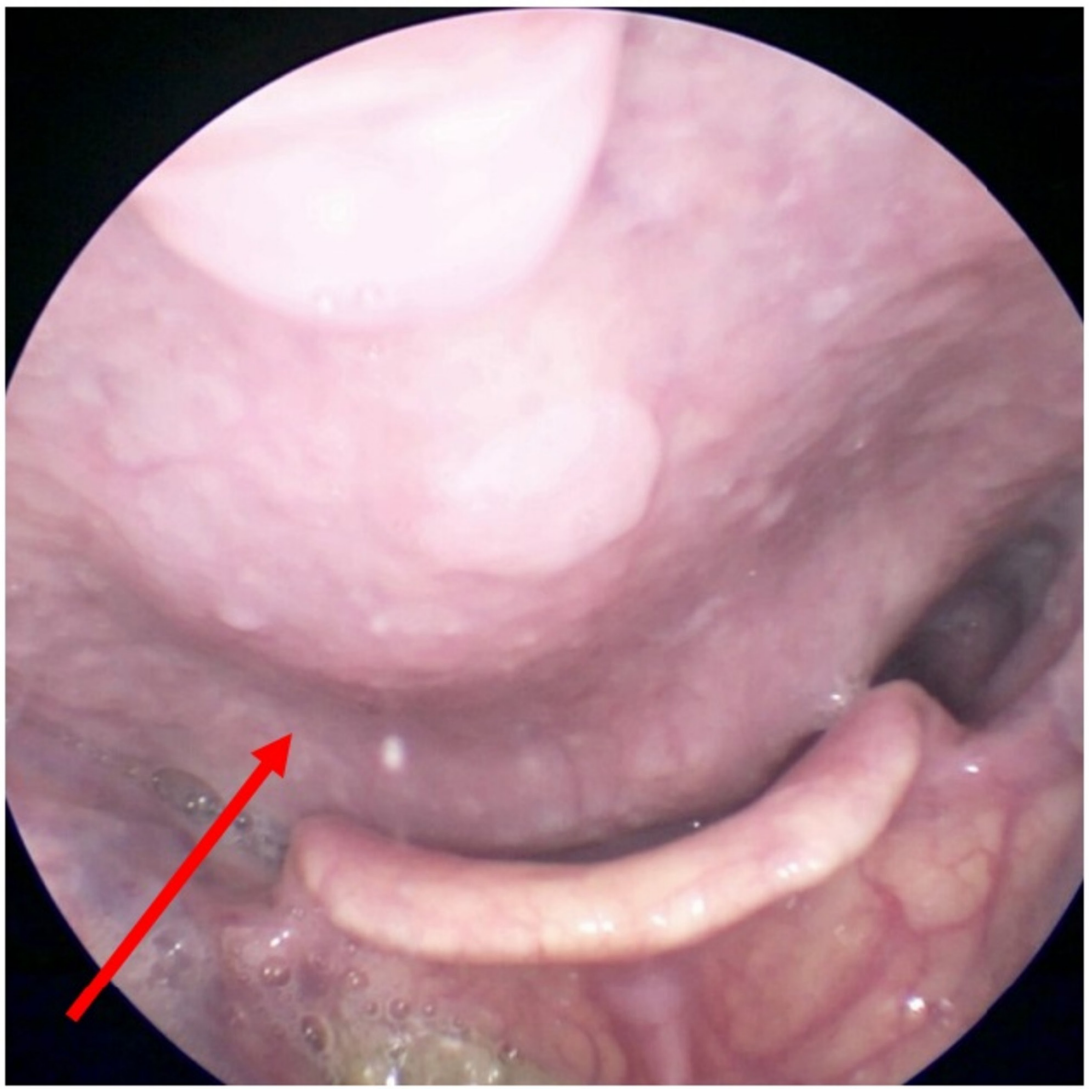
The larynx‐goscopic findings of Case 2. A significant bulge in the posterior larynx‐pharyngeal wall with a small papilloma is shown.

The second patient was a 70‐year‐old man seen from May 29 to June 1, 2019, with complaints of snoring during sleep for 20 years. The anthropometric data and physical examination results are shown in Table 1. The patient had no history of chronic heart disease, hypertension, type 2 diabetes, or other chronic diseases. UACT showed results as follows: (1) Obvious osteophytes were seen at the anterior edge of the cervical vertebra, (2) Compression and protuberation of the posterior pharyngeal wall were seen from the nasopharynx, oropharynx and posterior cricoid region, (3) The sagittal diameters of the relative pharyngeal cavity become narrower (Figure 2). The laryngoscopic findings revealed a significant bulge in the posterior laryngopharyngeal wall with small papilloma (Figure 3). The patient denied any ostealgia or other neurological symptoms and had no history of medical intervention or treatment for this condition. The PSG results (see Table 2) showed that the AHI was 72.5/h and the SAHI was 74.4/h. The minSat% was 81%. The pressure titration was also performed, and the 90% treatment pressure of CPAP was 12 cmH_2_O.

The Epworth Sleep Scale (ESS) score of these two cases was 8 and 13, respectively. Good compliance with the CPAP treatment and the significant improvement of subjective symptoms were reported from both patients after treatment. The post‐treatment AHI decreased to 11/h and 8.8/h, respectively. And the CPAP treatment was recommended by the physician. After leaving the hospital and returning home, both patients used CPAP regularly and have been receiving regular follow‐up.

## 3 DISCUSSION

These two male patients were both diagnosed with severe OSA. No typical physical findings, such as an elevated tongue position, obvious jaw recession or a high BMI, were found in the outpatient department. The most positive findings were anatomical abnormalities of the cervical spine, which inspired us to further explore their correlation.

We searched the literature for papers on OSA and cervical spine disorder. The progression of OSA was associated with a history of cervical fusions and osteophytes. Cervical spondylosis was reported as a rare risk factor for OSA.^1^ Sonnesen et al.^2^ recruited 57 OSA patients in Denmark, 21.1% of whom were found to have coexistent cervical spine fusion abnormalities. Pham et al.^3^ tried to examine the association between OSA and cervical spine pathologies, postural changes, and pain, and they speculated that the mechanism might be as follows: (1) Fusion of the cervical spine reduces the retropharyngeal space. (2) Bone spurs or osteomas aggravate the retropharyngeal compression. (3) Decreased cervical compliance and mobility aggravate the OSA severity.^2^ Cervical forward bending and head extension were also reported as significant independent risk factors for OSA severity.^4^

In this case report, both patients had high AHI, SAHI, and CPAP therapeutic pressures and no other positive physical findings, which gave us the reason to speculate that their OSA condition was related to cervical spine anatomy abnormalities. Cervical spine mobility caused by cervical internal fixation and spinal osteophytes reduced the space of the upper airway. We also speculate that surgeries of improving the anterior cervical protrusion or cervical hyperplasia may be able to reverse the OSA condition. Well‐designed prospective studies are needed to validate the roles of these changes and guide otolaryngologists to propose more targeted treatment plans when treating OSA patients with combined cervical spine lesions. Furthermore, it is hoped that the characteristics of both cases may draw the attention of orthopedic surgeons, especially spine surgeons, to confirm whether their patients have any complaints of snoring or sleep apnea before and after surgery. An overnight PSG test could be suggested before and after the spine surgery. Chung et al.^5^ included OSA patients who underwent cervical or thoracic spine surgery. High probabilities of comorbidity, difficulties with postoperative recovery, high risks of pulmonary embolism and deep vein thrombosis were observed in patients with history of diseases or past surgical operations that could seriously affect cervical spine compliance and mobility. The evaluation of OSA would be essential to avoid adverse impacts on sleep breathing, which might yield more positive therapeutic outcomes.

## 4 CONCLUSION

Cervical spine anatomy abnormalities may contribute to the progression of OSA and could even be an independent risk factor of OSA onset in patients without any other risk factors. The combined occurrence of OSA and cervical spine disease requires joint attention from otolaryngologists and spine surgeons.

## AUTHOR CONTRIBUTIONS

Anrong Sun provided the idea of the case report, patient enrolment, and drafted the manuscript. Rui Fang involved in patient enrolment and revising of the manuscript. Huiping Luo involved in patient enrolment, provided the idea, and revised the manuscript.

## FUNDING INFORMATION

The study was supported by The Youth Foundation of Shanghai Municipal Health Commission (No. 20194Y0190).

## CONFLICT OF INTEREST

The authors have no conflict of interest to declare.

## ETHICAL APPROVAL

The study was approved by the Ethics Committee of the Eye & ENT Hospital, Fudan University (No. 2021101).

## CONSENT

Written consent was obtained from both patients prior to the writing of the case report.

## Data Availability

Data openly available in a public repository that issues datasets with DOIs.
